# Conditional entropy in variation-adjusted windows detects selection signatures associated with expression quantitative trait loci (eQTLs)

**DOI:** 10.1186/1471-2164-16-S8-S8

**Published:** 2015-06-18

**Authors:** Samuel K Handelman, Michal Seweryn, Ryan M Smith, Katherine Hartmann, Danxin Wang, Maciej Pietrzak, Andrew D Johnson, Andrzej Kloczkowski, Wolfgang Sadee

**Affiliations:** 1 Center for Pharmacogenomics, The Ohio State University College of Medicine, Graves Hall, 330 W. 10th Ave., Columbus, OH 43210, USA; 2 Mathematical Biosciences Institute, Jennings Hall 3rd Floor, 1735 Neil Ave., Columbus, OH 43210, USA; 3 Faculty of Mathematics and Computer Science, Łódź University, Narutowicza 65, 90-131 Łódź, Poland; 4 Division of Biostatistics, The Ohio State University College of Public Health, Cunz Hall, 1841 Neil Avenue, Columbus, OH 43210-1240, USA; 5 Division of Intramural Research, National Heart, Lung and Blood Institute, Cardiovascular Epidemiology and Human Genomics Branch, The Framingham Heart Study, 73 Mt. Wayte Ave., Suite #2, Framingham, MA, USA; 6 Battelle Center for Mathematical Medicine, Nationwide Children's Hospital, 700 Children's Drive, Columbus OH 43205, USA; 7 Department of Pediatrics, The Ohio State University College of Medicine, 700 Children's Drive, Columbus OH 43205, USA; 8 Kavli Institute for Theoretical Physics China, Chinese Academy of Sciences, Beijing 100190, China

**Keywords:** Haplotype, Positive Selection, Selective Sweep, Conditional Entropy, eQTL, Conditional Logistic Regression

## Abstract

**Background:**

Over the past 50,000 years, shifts in human-environmental or human-human interactions shaped genetic differences within and among human populations, including variants under positive selection. Shaped by environmental factors, such variants influence the genetics of modern health, disease, and treatment outcome. Because evolutionary processes tend to act on gene regulation, we test whether regulatory variants are under positive selection. We introduce a new approach to enhance detection of genetic markers undergoing positive selection, using conditional entropy to capture recent local selection signals. Results We use conditional logistic regression to compare our Adjusted Haplotype Conditional Entropy (H|H) measure of positive selection to existing positive selection measures. H|H and existing measures were applied to published regulatory variants acting in *cis *(*cis*-eQTLs), with conditional logistic regression testing whether regulatory variants undergo stronger positive selection than the surrounding gene.

These *cis*-eQTLs were drawn from six independent studies of genotype and RNA expression. The conditional logistic regression shows that, overall, H|H is substantially more powerful than existing positive-selection methods in identifying *cis-*eQTLs against other Single Nucleotide Polymorphisms (SNPs) in the same genes. When broken down by Gene Ontology, H|H predictions are particularly strong in some biological process categories, where regulatory variants are under strong positive selection compared to the bulk of the gene, distinct from those GO categories under overall positive selection. . However, *cis*-eQTLs in a second group of genes lack positive selection signatures detectable by H|H, consistent with ancient short haplotypes compared to the surrounding gene (for example, in innate immunity GO:0042742); under such other modes of selection, H|H would not be expected to be a strong predictor.. These conditional logistic regression models are adjusted for Minor allele frequency(MAF); otherwise, ascertainment bias is a huge factor in all eQTL data sets. Relationships between Gene Ontology categories, positive selection and eQTL specificity were replicated with H|H in a single larger data set. Our measure, Adjusted Haplotype Conditional Entropy (H|H), was essential in generating all of the results above because it: 1) is a stronger overall predictor for eQTLs than comparable existing approaches, and 2) shows low sequential auto-correlation, overcoming problems with convergence of these conditional regression statistical models.

**Conclusions:**

Our new method, H|H, provides a consistently more robust signal associated with cis-eQTLs compared to existing methods. We interpret this to indicate that some cis-eQTLs are under positive selection compared to their surrounding genes. Conditional entropy indicative of a selective sweep is an especially strong predictor of eQTLs for genes in several biological processes of medical interest. Where conditional entropy is a weak or negative predictor of eQTLs, such as innate immune genes, this would be consistent with balancing selection acting on such eQTLs over long time periods. Different measures of selection may be needed for variant prioritization under other modes of evolutionary selection.

## Background

In the post-genome era, major research initiatives are directed towards identifying non protein-coding genetic variants with an effect on the expression of nearby genes: *cis*-expression quantitative trait loci (*cis*-eQTLs) [1,2]. In parallel, comparative analysis [3] of humans and other primates, as well as human populations, has revealed categories of genes evolving under different selective pressures [4,5]. For example, the phylogenetic p-values program phyloP [6] has been used to show that disease markers in genes involved in central nervous system (CNS) developmental defects [7] have undergone positive selection in the human lineage. Humans have more fixed genetic differences in CNS development genes, compared to our close primate relatives; therefore, phyloP identifies mutations in these CNS development genes. The interpretation is that these mutations conferred differential fitness in the primate ancestors of modern humans, causing many mutations in such genes to rapidly accumulate and fix. Conversely, other genes and especially regulatory regions show human-lineage specific purifying selection [8], leading to a loss of ancestral diversity. Therefore, it might be expected that different selection measures would be more or less useful in different genes or regulatory features.

In this report, we explore positive selection signatures not in disease genes/markers arising from our pre-human ancestors, but in the *cis*-eQTLs within/adjacent to genes. Positive selection in this report is confined to a relatively recent timeframe, and is controlled for the human population positive selection level on the entire gene and sub-gene location (exon, intron, intergenic region, flanking within 1kB of promoter or terminator, or untranscribed region/UTR). The positive selection signals used here are measured using allele frequencies and linkage-related properties within and between human populations via the results of the 1,000 Genomes Project [9]. Detecting a controlled association between positive selection and *cis*-eQTLs is technically challenging from a statistics standpoint, because it introduces considerable dependencies (co-linearity) between the prediction (positive selection, allele frequency, sub-gene region) and stratification (gene) variables. The effort is justified because eQTLs, both cis [10] and trans [11] have shown considerable relevance to human disease. Methods to prioritize such variants have considerable practical consequences, since most disease heritability is driven by regulatory markers outside of coding regions [12,13].

In order to fit statistical models of *cis*-eQTLs within genes, we introduce a new measure of positive selection, termed Adjusted Conditional Haplotype Entropy (H|H). Conditional entropy is commonly used in information/linguistics approaches [14], but also sees use in diverse bioinformatics applications [15,16]. Among existing measures, this conditional entropy is most closely related to the integrated haplotype score (iHS) of Voight *et al*. [17], however, in the results below we will show that H|H provides two crucial advantages. First, H|H is a significantly stronger predictor of *cis-*eQTLs than other measures, and second, H|H values can vary widely even between nearby markers. H|H is a measure of haplotypes but it depends on an individual marker: for example, a minor variant leading to a selective sweep of the pre-existing major haplotype would result in a very low conditional entropy and thus a very high H|H score, but it would be in linkage to other major haplotype markers that would have very low H|H scores. This divergence between nearby markers and even markers in moderate linkage allows for models incorporating H|H to converge when models utilizing other positive selection measures do not.

## Results and discussion

### Overall research design

**Figure 1:**
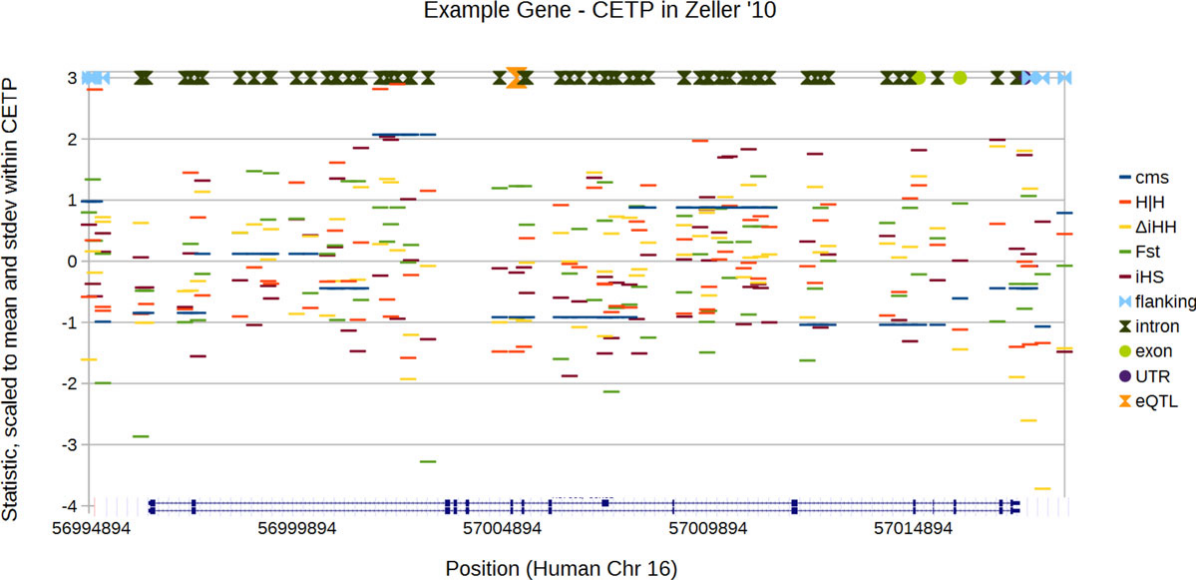
****Example showing positive selection predictors and eQTLs for a single gene****. This figure shows the input to the conditional logistic regression associated with a single gene (Cholesterol Ester Transfer Protein, or CETP) from the Zeller 2010 data set. The purposes of this figure are to illustrate the prediction problem in a single gene/strata, and to showcase the relative degree of serial auto-correlation (smoothness) associated with the different predictors. At the top of the figure, each SNP is indicated with a symbol reflecting the location within the gene, only one, rs1532625, is an eQTL in the Zeller data set (indicated with a large orange hourglass symbol), and it happens to be in an Intron; the two major splice isoforms of CETP are illustrated at the bottom of the figure for reference. rs1532625 does NOT show any particular sign of being under positive selection. The conditional logistic regression used in this manuscript is fit to eQTLs such as rs1532625, with genes such as CETP treated as individual strata (equivalent to a 1-to-many case-control matching in a clinical trial.) In this data set CETP contains only a single eQTL but this is not always the case. Four predictors used in this manuscript are scaled to empirical Z-scores in order to fit on the same chart; a fifth potential predictor (composite of multiple signals, cms), very powerful in other contexts, is also shown to illustrate the issue with auto-correlation. Conditional logistic models depend on a degree of independence among the predictors - because the cms score (blue line) has such a strong serial auto-correlation (as would any positive selection measure that is smoothed in a window of any size), it is not independent of the within-gene location (symbols at the top) which are used as an independent predictor. Even Fst (the green line) shows too much serial auto-correlation to converge in the Mangravite data set, which was part of the motivation in developing H|H. The other three positive selection measures, including H|H, are highly non-smooth, so they can be fit to logistic models where individual strata contain short regions of DNA.

The chief goal of this manuscript is to identify measures of positive selection which are able to pick out cis-eQTLs, against a background of non-eQTL markers within an individual gene. However, such measures of positive selection are not uniform throughout the genome. In particular, positive selection signatures are concentrated in certain gene ontology (GO) [18] categories. Therefore, different measures of positive selection may be appropriate to pick out eQTLs in different GO categories, and this appropriateness could easily be counter-intuitive - genes under positive selection could have a high background level of positive selection, meaning positive selection measures might not be useful in picking out eQTLs within genes which are highly selected overall. Therefore, the secondary goal of this manuscript is to identify differences in which positive selection measures may be most appropriate to identify eQTLs in different GO categories. To test for such difference, we add interaction terms our models; this is not a GO enrichment test. GO enrichment tests implemented in panther [19] are also reported in the supplement (in additional file 1), but these may not have the desired interpretation. This manuscript also reports a new measure of positive selection which is designed to support this application, chiefly by being as non-smooth as possible (and thus able to distinguish eQTLs from non-eQTLs which are close by on the chromosome.) Figure 1 illustrates the underlying problem: the goal is to find a statistical model which uses positive selection measures to distinguish eQTLs (the one orange hourglass, in Figure 1) from all of the non-eQTL markers in the same gene (all other symbols). The conditional logistic regression adjusts for the relevative prevalance of eQTLs in different genes (for example, the gene shown in Figure 1 only has one eQTL in the Zeller 2010 data set.)Our statistical approach has two phases. In the first (hypothesis-generation) phase, seven modestly-powered QTL data sets from either Lymphoblastic Cell Lines (LCLs), Monocytes or human liver [20-25] are used to pick prediction variables, and, categories of genes in which positive selection scores differentiate *cis*-eQTLs from the surrounding gene. In the statistical tests of this hypothesis-generation phase, other measures of positive selection where either not strong predictors of eQTLs; or, attempts to utilize measures other than H|H failed because the statistical models did not converge. However, because we cannot guarantee that the statistical model generated by this first stage is not subject to over-fitting, or alternatively to cherry-picking, we test that statistical model in a second phase. In this second (hypothesis-testing) phase, we test specific biological findings in the much larger *cis*-eQTL data set which powers the blood eQTL browser described by Westra *et al*. [11].

In both phases, conditional logistic regression is used to fit statistical models to the different *cis*-eQTL data sets. GO categories and other measures individually identified in the hypothesis-generation phase are tested as a group in the hypothesis-testing phase.

### Hypothesis-testing phase in the Chicago QTL Browser data

For purposes of hypothesis generation, seven diverse eQTL data sets (drawn from six independent high-throughput studies) were chosen from those deposited in the Chicago QTL genome browser [26] (http://hsb.upf.edu/), and reduced to their overlap with the set of Genotype-Tissue Expression (GTEx) [27] candidate QTLs. These candidate QTLs are a superset including all GTEx QTLs as well as other non-QTL markers for which GTEx performed statistical tests; these are chosen by GTEx as a representative panel to test for effects, and not because of prior evidence that they are indeed eQTLs. Genes (or intergenic regions, defined here as more than 1kB from any promoter or transcriptional terminator) containing no eQTLs were removed independently in each data set. The focus is on hypothesis generation, parameter selection and model specification, rather than on producing a rigorous finding. These seven eQTL data sets were chosen because they contained at least 10,000 eQTLs after overlap with the GTEx candidates. The seven data sets chosen are listed in Table 1 along with summary statistics of those data sets after they were reduced to the overlap with the GTEx set of candidate QTLs.

**Table 1:** Chicago QTL browser data sets.

Dataset	Notes	Number of eQTLs/total	Num. Genes (Regions)	Total GTEx Markers
Mangravite 2012 [20]	LCLs; candidate genes	42,550/49,937	2,064 (2,030)	385,836

Montgomery 2010A [21]	LCLs; RNA-seq; exons	12,691/13,965	2,755 (2,155)	1,389,595

Montgomery 2010B [21]	LCLs; RNA-seq; transcripts	3,881/4,748	934 (706)	493,074

Schadt 2007 [22]	Liver	2,558/2,694	1,523 (857)	862,416

Stranger 2007 [23]	LCLs	12,751/15,067	1,309 (961)	289,863

Veyrieras 2008 [24]	LCLs	13,730/15,939	1,437 (1016)	296,696

Zeller 2010 [25]	Monocytes	36,371/39,923	5,532 (3,931)	1,398,426

For each **Dataset **with primary reference: **Notes **on the sample in which eQTLs were measured; the **Number of eQTLs **below their significant threshold and which are also present in the GTEx candidate panel (out of the total number of QTLs passing the threshold in the source data set); the **Number of Genes **(and intergenic Regions, each of which was analysed as if a gene) which contained at least one eQTL present in both data sets; and, the **Total GTEx Markers **present in those genes, whether they are eQTLs in the corresponding data set or not.

When statistical models were fit to these individual data sets, the results were quite heterogeneous (see below in this section). Therefore, GO biological-process categories were used to test whether the inter-data set heterogeneity might be explained by differences in the underlying biology of the genes for which eQTLs were detected by the different approaches. Alternative, non-exclusive explanations for heterogeneity among these data sets include: source tissue differences, participant population differences, and sample size/ascertainment bias differences, any or all of which might reasonably affect the extent to which eQTLs appear more positively selected than nearby markers. In order to produce a preliminary hypothesis on this question, each gene was assigned to a single biological-process GO category, chosen among the available GO assignments (see methods), with genes assigned to multiple GO categories assigned to the single GO category containing closest-to-125 ENCODE [28] genes. This is an *ad hoc *approach that produces single assignments to biologically relevant GO categories; it was driven by inspection of the GO categories of most interest to our research group. Size-filtering of some kind is standard in any study of GO categories[29]. Note that this is not a GO enrichment test, and is neither intended nor expected to give a valid GO enrichment result, but to test for an interaction between GO category and our QTL predictors. So, for example, these results cannot be interpreted to indicate in which GO categories QTLs are especially common. The reduction to a single GO assignment for each gene is both a simplifying assumption and a technical requirement for the type of model fitting that is used in the hypothesis-generation phase; the GO category size of 125 was chosen by inspection - GO categories containing more than 200 genes were generally not biologically informative (*e.g*. the most extreme case, GO 0008150, *biological process*), while GO categories below size 50 are unlikely to have enough representatives in our data to detect an association, especially an association which is reproduced in several data sets containing a non-overlapping combination of genes. Small GO categories are often not-represented at all in many of our data sets. This procedure will be biased to detect effects from larger GO categories, but the statistical test should still be valid, in the same way that a non-uniformly distributed error rate (*e.g*. in a GWAS, assigning 50% of the errors to the 5% of the markers which are in coding regions)

This may introduce a bias into the types of GO categories which are selected in this hypothesis-generation phase; yet another reason why the hypothesis-testing phase was required. The GO assignment reduction is not carried out in the hypothesis-testing phase, so the hypothesis-testing phase should be free of any bias introduced by this favoritism.

In each of the seven data sets, conditional logistic regression was used to fit six statistical models, each with gene/intergenic region as a stratification variable, which are given in Table 2.

**Table 2:** Conditional logistic models.

Model	Formula
1	eQTL ~ H|H + |ΔDAF| + ΔDAF + ΔiHH + F_st _+ iHS + MAF + location

2	eQTL ~ H|H + |ΔDAF| + ΔDAF + ΔiHH + iHS + MAF + location

3	eQTL ~ H|H + |ΔDAF| + ΔDAF + ΔiHH + F_st _+ iHS + MAF × GO + location

4	eQTL ~ H|H + |ΔDAF| + ΔDAF + F_st _+ MAF × GO + location

5	eQTL ~ H|H + |ΔDAF| + ΔDAF + MAF × GO + location

6	**eQTL ~ H|H × GO + ΔDAF + MAF × GO + location**

Each of the formulas above is fit to an intercept (α) and to one or more effect sizes (β, one associated with each term).

Except for our Adjusted Haplotype Conditional Entropy (H|H), all of the values in the formulas for Table 2 are taken from the 1,000 Genomes Selection Browser[26], and converted to a sum of logs (P value when reported, but MAF is similarly transformed although it is not a p-value) across thousand genomes populations on a per-SNP basis. Identification as an eQTL is treated as a random variable, with a logistic distribution fit on a per-gene basis to an effect from one or more of the following variables that are included in the formulas in Table 2: Adjusted Haplotype Conditional Entropy (H|H, the new measure introduced in this manuscript), the absolute value of the differential allele frequency (|ΔDAF|[30]), the differential allele frequency (ΔDAF[30]), the change in integrated haplotype heterozygosity (ΔiHH[17]), the coancestry coefficient (F-statistic or F_st _[31]) the integrated haplotype score (iHS[17]), the minor allele frequency (MAF), and the location within the gene of an individual SNP (either flanking, UTR, intronic or exonic; or, for intergenic regions, intergenic); if an individual parameter is fit independently in each singletized GO term, it is appended to indicate such an interaction (with × GO).

The results of the fits for the formulas in Table 2 are given in **Table S1 **(in the supplement, additional file 1). The parameter values in **Table S1 **are summarized and interpreted in the next few paragraphs, and typical values for the Z-scores of the individual components are given in Table 3. As shown in Table 3 H|H is generally a much stronger predictor than other positive selection measures, with much higher Z-scores of consistent sign; ΔiHH and iHS are both weaker predictors, and the relationship between the sign of the value and whether or not a marker is an eQTL actually changes between data sets.

**Table 3:** Summary of t-statistic Z-scores from conditional logistic model fits

Dataset	Z-score H|H	Z-s. |ΔDAF|	Z-s. F_st_	Z-s. ΔiHH	Z-s. iHS
Mangravite 2012	22	6/NA	NA	0	8

Montgomery 2010A	13	3/5	-4	1	-2

Montgomery 2010B	8	2/4	-3	3	-2

Schadt 2007	2	1/1	-1	1	0

Stranger 2007	8	-2/4	-8	-3	-2

Veyrieras 2008	8	0/0	-13	-3	-1

Zeller 2010	19	2/7	-7	2	0

For each **Dataset**, the average value of the Z-score across all Models (1-5 from Table 2, rounded to the nearest whole number.) Two averages are given |ΔDAF| - Models 2 and 5 before the slash; Models 1, 3 and 4 after the slash. For H|H, a positive number indicates a positive value is a strong predictor for eQTLs, for the other measures, an extreme negative value indicates that a low log p-value is a strong predictor for eQTLs. ΔiHH and iHS are intended to be measures of positive selection. With the exception of |ΔDAF| (which changes substantially depending on whether F_st _is included in the model), these Z-scores do not change greatly among Models 1-6 in Table 2, indicating that these predictors are largely independent.

Minor allele frequency is the strongest predictor, and heavily modulates the predictive power of all other predictors (results not shown; this is an expected consequence of sample size constraints/ascertainment bias within the individual source studies.) Otherwise, only one pair of predictors interact (change substantially in predictive power when both are included): |ΔDAF| and F_st_. For |ΔDAF|, two Z-scores are given in Table 3: without (before the slash) and with (after the slash) F_st _in the model. F_st _is a good predictor but does not converge on the Mangravite data set, indicative that the measure is overly smooth (see Figure 1).

Model 6 is underlined in the list above because it is used for hypothesis generation; Model 6 includes only our Adjusted Haplotype Conditional Entropy (fit on a per GO term basis), Minor Allele Frequency (likewise), and Differential Allele Frequency (fit to each data set as a whole). Other positive selection measures either were weak predictors in one or more data sets (ΔiHH and iHS; |ΔDAF| unless F_st _is also included); or, caused a failure of the model to converge in some data sets (F_st_, ΔDAF if included as an interaction term), because of excessive non-independence (referred to as co-linearity in the logistic regression literature [32]) with either the other predictors or with the stratification variable. ΔDAF is a strong predictor and we were able to include ΔDAF in models that did converge; ΔDAF serves a crucial purpose in our models because it differentiates the effects of population bottlenecks from selective sweeps. However, unfortunately a model including a ΔDAF interaction with GO terms was tried but did not converge, so ΔDAF was fit globally. Notably, of the positive selection measures, only our Adjusted Haplotype Conditional Entropy is a strong predictor which also converges in all of these data sets when fit on a per-GO term basis.

Other positive selection measures that report values for contiguous blocks of gene sequence are highly useful in gene-prioritization applications[33]; however, they are generally unsuited for the challenge addressed here, which is to prioritize variants within genes, and would be expected to have severe convergence issues, so they were not considered (Figure 1 illustrates this issue with the Composite of Multiple Signals[34]). Future applications of this method would be in a candidate gene context, however in order to assess the predictive power of the measure, whole genome results are used. A positive selection measure that is constant across an individual gene may identify genes that are in turn eQTL rich, but this would tend to produce a failure to converge. In any case, in the currently available data, differences in eQTL richness between genes cannot be distinguished from ascertainment bias. The conditional information haplotype score used here was developed in part to address these technical issues, which are related to the initial observation made by biologists in our group - that a marker in a long haplotype might be only a few hundred bases away from a second marker with which the long haplotype is in weak linkage. In these data, only our new conditional haplotype information has the properties that it is both a strong predictor for eQTLs and allows a convergent statistical fit in relevant cases. Therefore, **Model 6 **was chosen to carry on to the hypothesis generation step.

### GO ID's at high and low extremes of selection score in hypothesis-generation data set

A heuristic score was used to combine the results of the fit to **Model 6**, for each GO ID in each of the seven data sets. To generate this heuristic score from the fit of **Model 6 **to each permutation, the effect size is moved one standard error towards 0 (minimum of 0) and then summed across all 7 data sets. There is an overall association between our H|H and eQTLs; by using the effect size rather than the Z-score, we avoid identifying individual GO IDs solely because they contain many genes.

In order to establish preliminary confidence bounds on the range of variability for these prediction by GO category interactions, **Model 6 **was also fit to 100 randomly permuted versions of the data sets above. In each permutation, assignments between genes assigned to each single, medium-sized GO category, were randomized. In each permutation, each randomized medium-sized GO category contained the same number of member genes as the corresponding original GO category. By generating the same heuristic score (sum of effect sizes) in each permutation, a confidence interval was obtained.

The effect sizes and standard errors for the GO categories outside of the 90% confidence interval (see **Meta-analysis of conditional logistic regression regression results **for details and justification of this overall approach) are given in Table 4. The following GO categories, hence **Group 1**, were below the 90% confidence interval from the permutations (that is, in these categories, overall the Conditional Haplotype Entropy was low for eQTLs compared to other GO categories):

**Table 4:** GO IDs where eQTL effect size for Adjusted Haplotype Conditional Entropy outside of the 90% confidence interval from permutation.

Gene Ontology	Mangravite 2012: β_H|H ± σH|H_	Montgomer y'10 Exon: β_H|H ± σH|H_	Montgomer y '10 Trspt: β_H|H ± σH|H_	Schadt 2007: β_H|H ± σH|H_	Stranger 2007: β_H|H ± σH|H_	Veyrieras 2008: β_H|H ± σH|H_	Zeller 2010: β_H|H ± σH|H_
Negative Effect Sizes							

GO:**0006367**	**-0.099 ± 0.055**	*0.186 ± 0.086*	-0.402 ± 0.592	0.137 ± 0.255	**-1.600 ± 1.200**	-0.001 ± 0.172	-0.048 ± 0.060

GO:**0006396**	**-0.112 ± 0.041**	**-0.066 ± 0.065**	**-0.207 ± 0.109**	-0.0001 ± 0.134	**-0.160 ± 0.069**	-0.049 ± 0.074	**-0.050 ± 0.035**

GO:**0008544**	*no genes*	-0.013 ± 0.125	*no genes*	**-1.200 ± 0.933**	*no genes*	-0.043 ± 0.126	0.032 ± 0.043

GO:**0042742**	**-0.373 ± 0.105**	**-0.140 ± 0.111**	-0.006 ± 0.249	-0.055 ± 0.180	-0.107 ± 0.123	-0.068 ± 0.132	0.019 ± 0.056

Large Positive Effect Sizes							

GO:**0001501**	**0.128 ± 0.047**	**0.158 ± 0.075**	**0.290 ± 0.083**	0.006 ± 0.127	0.196 ± 0.330	**0.139 ± 0.094**	**0.039 ± 0.039**

GO:**0006869**	**1.144 ± 0.643**	**0.119 ± 0.069**	*-0.037 ± 0.011*	-0.074 ± 0.124	0.455 ± 0.676	0.066 ± 0.100	0.018 ± 0.037

GO:**0006936**	**0.480 ± 0.076**	*-0.124 ± 0.052*	-0.052 ± 0.116	0.084 ± 0.109	**0.629 ± 0.262**	**0.304 ± 0.082**	0.010 ± 0.030

GO:**0007186**	**0.657 ± 0.452**	**0.309 ± 0.209**	0.090 ± 0.259	*no genes*	**0.771 ± 0.319**	-0.143 ± 0.231	-0.067 ± *0.108

GO:**0009653**	0.010 ± 0.033	0.040 ± 0.061	**0.414 ± 0.194**	0.084 ± 0.100	**0.322 ± 0.097**	**0.060 ± 0.044**	**0.037 ± 0.035**

GO:**0016567**	**0.185 ± 0.042**	0.040 ± 0.071	-0.031 ± 0.0112	*-0.322 ± 0.239*	**0.297 ± 0.059**	**0.113 ± 0.047**	0.038 ± 0.040

GO:**0018108**	**0.277 ± 0.049**	0.027 ± 0.086	0.048 ± 0.094	0.031 ± 0.128	**0.465 ± 0.119**	**0.175 ± 0.078**	0.026 ± 0.026

GO:**0051056**	**0.185 ± 0.044**	0.030 ± 0.055	-0.006 ± q0.173	-0.136 ± 0.169	**0.218 ± 0.103**	**0.333 ± 0.111**	-0.033 ± 0.039

Each row corresponds to GO ID, and each cell gives the logistic-scale effect size (β) and standard error (σ), for the Adjusted Haplotype Conditional Entropy (H|H) for eQTLs in genes within that GO category from a conditional logistic regression under Model 6. A bold cell indicates a term that is contributing to membership in the corresponding group; a red italicized cell indicates a term that is pointing in the opposite direction.

http://amigo.geneontology.org/amigo/term/GO:0006367 transcription initiation from RNA polymerase II promoter

http://amigo.geneontology.org/amigo/term/GO:0006396 RNA processing

http://amigo.geneontology.org/amigo/term/GO:0008544 epidermis development

http://amigo.geneontology.org/amigo/term/GO:0042742 defense response to bacterium

While the following GO categories, hence **Group 2**, were above the 90% confidence interval from the permutations (that is, in these categories, overall the Conditional Haplotype Entropy, which is generally higher for eQTLs, was especially-relatively-high for eQTLs compared to other GO categories):

http://amigo.geneontology.org/amigo/term/GO:0001501 skeletal system development

http://amigo.geneontology.org/amigo/term/GO:0006869 lipid transport

http://amigo.geneontology.org/amigo/term/GO:0006936 muscle contraction

http://amigo.geneontology.org/amigo/term/GO:0007186 G-protein coupled receptor signaling pathway

http://amigo.geneontology.org/amigo/term/GO:0009653 anatomical structure morphogenesis

http://amigo.geneontology.org/amigo/term/GO:0016567 protein ubiquitination

http://amigo.geneontology.org/amigo/term/GO:0018108 peptidyl-tyrosine phosphorylation

http://amigo.geneontology.org/amigo/term/GO:0051056 regulation of small GTPase mediated signal transduction

**Group 1 **includes bacterial defense genes which might be expected to experience balancing selection[35,36], which would preserve ancient diversity, allowing time for recombination to produce short haplotypes; since the epidermis serves a largely infection-defense role, we speculate that balancing selection also acts there in order to maintain within-population diversity in pathogen environment. For the other categories in **Group 1**, we propose simply that the last 50,000 years is too short a time frame for many beneficial mutations to arise in the highly-conserved core functions of molecular biology[37].

**Group 2 **is of particular interest because it includes so many current and emerging drug targets[38,39], as well as the lipid transport genes which play such a large role in western lifestyle diseases[40,41]. Follow-up work will focus on utilizing these predictions to enrich the variant discovery activities of the Pharmacogenomics Research Network (PGRN)[42].

An additional GO term (Cellular Metabolic Process, GO:0044237) was also above the confidence interval but we chose to exclude it from the hypothesis-testing phase, because by our approach it produces a conglomerate taxon of genes that are simply not well-understood enough to be assigned to any GO ID with fewer members. This is not intended to be a rigorous statistical test of these associations; instead, these two categories are tested in a larger, more extensively genotyped hypothesis-testing phase, described next.

### Hypothesis-testing phase in the Westra data set/Blood eQTL Browser

The following variant of **Model 6 **was then fit to a single, substantially larger data set of eQTLs from whole blood, as a hypothesis-testing phase:

(1) eQTL~H|H×Group+ΔDAF+MAF×Group+location

This hypothesis-testing differed from **Model 6 **in the hypothesis-generation phase above in that:

1) intergenic regions (defined as more than 1kB from an adjacent gene) were associated with GO IDs, and thus groups, of both their flanking genes,

2) all genes associated with the GO IDs in **Group 1 **and/or **Group 2 **were included (and not just those that were not also assigned to a second GO ID which had closer-to-125 members), in order to avoid any biases that might have been introduced by our size-filtering,

3) the interaction terms were fit to each of **Group 1 **and **Group 2 **(and independently to the few genes and intergenic regions that were a member of GO IDs in both),

4) the data set itself includes more eQTLs as a fraction of all markers, including eQTLs having effect sizes too small to be detected in the Chicago eQTL browser data sets used for the hypothesis-generation phase.

In this test, shown in Table 5**Group 1 **and **Group 2 **do show statistically significant differences in the effect size for our Conditional Haplotype Entropy (H|H) measure (two-tailed P < 0.012 extrapolated from the standard error of the two t-test results from the conditional logistic regression). This confirms the biological findings outlined in the previous section, although **Group 1 **eQTLs shows no association with H|H at all (rather than a negative association) and the association in **Group 2 **is somewhat smaller than it was in the hypothesis-generation phase data. These differences may be attributable to some degree of cherry-picking in the hypothesis-generation phase (Group 2 would be expected to include GO IDs in which the effect size for H|H appeared strong by chance, implying that there are many more categories of GO IDs for which a significant difference in H|H effect size exists), or may imply that H|H is a predictor only for strong eQTLs (*i.e*. having a large effect on expression), which are in turn readily detected in the smaller data used in the validation phase above but swamped by weaker eQTLs in the larger validation data set. These possibilities will need to be distinguished in future work.

**Table 5:** Whole-blood *cis*-eQTL conditional logistic regression results

Dataset	Notes	Number of *cis*-eQTLs	Num. Genes (Regions)	Total GTEx Markers	βH|H ± σH|H
Westra 2012 - Group 1	GO:0006367, GO:0006396, GO:0008544, GO:0042742	11,527	324 (441)	108,372	-0.003 ± 0.003

Westra 2012 - Group 2	GO:0001501, GO:0006869, GO:0006936, GO:0007186, GO:0009653, GO:0016567, GO:0018108, GO:0051056,	63,269	1,265 (1,810)	598,118	0.005 ± 0.001

Westra 2012 - Both	In both Group 1 and in Group 2	1,673	17 (57)	18,447	0.006 ± 0.007

Westra 2012 - TOTAL	RNA extracted from whole blood.	495,268 /923,021	15,742 (14,417)	4,606,410	

As Table 1 with two additional columns reporting the effect size (β) and standard error (σ) of Adjusted Conditional Haplotype Entropy (H|H) in the hypothesis-testing phase fit to the statistical model in **Eq. 1 **using the Westra[11] data set.

#### GO enrichment test on normalized likelihoods

The previous results arise from incorporation of interaction terms into regression methods, and do not utilize existing approaches for GO enrichment. In the supplement (additional file 1), we present the results of an alternative approach where positive selection methods are added to the logistic model, the improvement in model prediction is assessed on a per-gene basis, and PantherDB is used to test for an association between model improvement and biological function GO category. This method finds GO categories containing relatively few genes and a significant interaction is not detected in the hypothesis-testing data set. A supplementary report on this alternative approach can be found in additional file 1.

## Conclusions

Our Adjusted Conditional Haplotype Entropy (H|H) will be distrubted as UCSC Genome Browser tracks[43] from the Center for Pharmacogenomics webpage (http://pharmacogenomics.osu.edu/). Conditional entropy indicative of a selective sweep is a strong predictor of eQTLs for genes in some medically interesting biological processes but not in others. In categories of genes where H|H is a weak or negative predictor of eQTLs, such as innate immune system genes, this would be consistent with balancing selection acting on such eQTLs. The evolutionary signatures underlying eQTLs in different biological processes, populations[44] and systems vary widely.

## Methods

### GTEx candidate SNPs

The GTEx consortium[27] has filtered human genetic variation for excess linkage disequilibrium, producing a list of 6,820,472 candidate variations for which GTEx plans to perform statistical tests in their tissues, which we downloaded from their ftp site on July 5, 2014.

First, because this list of SNPs has been filtered for D'[45] values, the risk of over-counting in our statistical model fits is reduced. Second, we anticipate that tissue-specific QTLs identified by GTEx will be the most relevant data for future applications that may incorporate selection as a component.

### Adjusted conditional entropy in 1,000 genomes data

For these calculations, 1,000 genomes data[9] release 3 (Nov. 23, 2010)[46] was used, including all single nucleotide polymorphisms (but not other types of variation) to which a reference SNP cluster (rs number)[47] had been assigned. Our method for finding positively-selected (long) haplotypes is based on information theoretical considerations, in particular the Khinchin axiom for the Shannon entropy, also known as the (Shannon) entropy decomposition formula[48]; for the purposes of this manuscript, the Khinchin axiom is used only to justify the summation of conditional entropy in different populations of roughly equal size. The formulas below describe the decomposition of the entropy for a population of haplotypes (in a given window), into a total entropy and a conditional entropy associated with a minor variant - the conditional entropy associated with the major variant would also be a part of this decomposition but is not used for our score. Frequency-dependent terms, the total entropy, the conditional entropy for the minor variant, and also any population-specific effects are all combined into an ad-hoc linear Adjusted Conditional Haplotype Entropy (H|H), which has a mean of 0 in each 1,000 genomes population.

The difference between the total entropy and the conditional entropy given the minor variant quantifies the influence of the minor variant on the diversity of the haplotypes in the window. The larger the difference between entropies, the more conserved (longer) the haplotype that carries the minor variant, consistent with a recent selective sweep.

First, independently in each population, a window is established around each marker SNP, m (where m must be a **GTEx Candidate SNP**, defined above, but the SNPs in the window are not so constrained):

(1A) $$\text{bpmin}:\left(\underset{\text{bpmin}\geq \text{i}>\text{m}}{\sum }{\text{H}}_{\text{i}}-10\right)\;\text{is minimized }$$

(1B) $$\text{bpmax}:\left(\underset{\text{m > i}\geq \text{bpmax}}{\sum }{\text{H}}_{\text{i}}-10\right)\;\text{is minimized }$$

(meaning: bpmin and bpmax are chosen such-that the sum is at least 10 but otherwise as small as possible).

Where H_i _is the Shannon entropy[49] at each SNP in the window (here using the natural log as the base) in the corresponding 1,000 genomes population. The sum of H_i _in the window would correspond to entropy of the window if the individual positions were all mutually independent. The value of 10 was chosen arbitrarily in order to make the results computationally tractable.

This gives a window that will be shorter around markers in more variable regions of the genome, and for the same markers will be shorter in more genetically diverse populations; this is intended to account for background differences in haplotype lengths between populations and regions.

Within this window, each 1,000 genomes chromosome is assigned to a haplotype, containing all the markers from bpmin to bpmax excluding the original marker m. Given that each haplotype J is observed in some fraction n*_J _of the 1,000 genomes participants in population p, we define H*p_m_:

(2) $$\text{H}*{\text{p}}_{\text{m}}=-\underset{\text{J}:\text{n}{*}_{\text{J}}>0}{\sum }\text{ln}\left(\text{n}{*}_{\text{J}}\right)\times \text{n}{*}_{\text{J}}$$

(this is the true entropy of the window - zero minus the sum over all haplotypes J in the population of the haplotype frequency times the log haplotype frequency). Further, given that each haplotype J is observed in some fraction of the n'_J _of the 1,000 genomes participants in population p who are **carrying the minor variant at m**, we define H'p_m_:

(3) $$\text{H'}{\text{p}}_{\text{m}}=-\underset{\text{J}:\text{n}{\text{'}}_{\text{J}}>0}{\sum }\text{ln}\left(\text{n}{\text{'}}_{\text{J}}\right)\times \text{n}{\text{'}}_{\text{J}}$$

Finally, these entropies are summed across all 1,000 genomes populations, and adjusted in order to account for the linear contribution of the log of the minor allele frequency (note that this factor also replaces the frequency weight which would apply for the true entropy decomposition) to give the Adjusted Haplotype Conditional Entropy (H|H); N_p _is the number of chromosomes in population p, N_Mp _is the number of major allele carrying chromosomes in population p, and _mp _is the number of minor allele carrying chromosomes in population p.

(4) $$\text{H|}{\text{H}}_{\text{m}}=-\underset{\text{all p}:{\text{N}}_{\text{Mp}}>5}{\sum }\text{H}*{\text{p}}_{\text{m}}-\text{H'}{\text{p}}_{\text{m}}-\alpha_{\text{p}}-\beta_{\text{Mp}}\text{ln}\left({\text{N}}_{\text{Mp}}\right)-\beta_{\text{mp}}\text{ln}\left({\text{N}}_{\text{mp}}\right)$$

α_p_, β_Mp_, and β_mp _are chosen empirically so that the average value of this sum, in each 1,000 genomes population, would be 0; see Table 6 below. The intent is that the adjusted conditional entropy would be more independent of both the minor allele frequency and the number of populations in which the SNP is found, both of which are better expressed using the minor allele frequency and differential allele frequency measures.

**Table 6:** Empirical adjustments in conditional entropy.

**Population**	**Α**	**β_m_**	**β_M_**
ASW	4.9	-0.74	-0.22
CEU	5.8	-0.54	-0.52
CHB	6.8	-0.50	-0.75
CHS	7.0	-0.51	-0.76
CLM	5.2	-0.54	-0.48
FIN	5.6	-0.53	-0.47
GBR	6.0	-0.56	-0.55
IBS	5.2	-0.81	-0.79
JPT	7.2	-0.56	-0.79
LWK	4.0	-0.64	-0.03
MXL	5.7	-0.49	-0.60
PUR	5.5	-0.58	-0.52
TSI	6.0	-0.53	-0.55
YRI	4.3	-0.65	-0.11

Each cell gives the paramaters for a regression fit across all GTEx candidate eQTLs for the Adjusted Haplotype Conditional Entropy (H|H) in the corresponding population, including the intercept (α), the contribution from the number of chromosomes carrying the minor variant (β_m_), and the contribution from the number of chromosomes carrying the major variant (β_M_).

### Chicago eQTL browser data sets

Via the Chicago eQTL browser (http://eqtl.uchicago.edu/cgi-bin/gbrowse/eqtl/) we obtained the seven QTL data sets listed in Table 1.

These data sets were used only in the hypothesis-generation phase.

### Westra/Blood eQTLs

Via the blood eQTL browser maintained by the authors (http://genenetwork.nl/bloodeqtlbrowser/) of a study[11] primarily focused on potential disease consequences for *trans*-eQTLs, we obtained *cis*-eQTLs from a very large (5,311 participants in **their **discovery phase, with an additional 2,775 participants in **their **replication phase) study of expression genetics in whole human blood. These *cis*-eQTLs are used only in **our **hypothesis-testing phase.

### 1,000 Genomes Selection Browser results

A variety of diversity and positive selection genetics measures are made available *via *the 1,000 Genomes Selection Browser[26]. The entire data set was downloaded on January 10, 2014.

Of these, ΔDAF[30], |ΔDAF|[30], ΔiHH[17], F_st _[31], and iHS[17], along with minor allele frequency (MAF) were used in at least one model in the hypothesis-generation phase. In the final model and in the hypothesis-testing phase, only ΔDAF and MAF were used. Positive selection measures that report results in windows (including the Composite of Multiple Signals[34] score, distributed elsewhere) were not used because preliminary results indicated that the gene-based **Conditional logistic regression **would not converge.

### Covariates incorporated using Annovar and the UCSC Table Browser

The results from the previous sections were matched to genes and within-gene location using Annovar[50] which in-turn utilizes 2013 ENCODE data[28]. Once mapped to gene symbols, gene ontology[18] IDs are mapped using the UCSC table browser[51] with tables downloaded on or after September 25, 2014.

To account for differential eQTL frequencies in different locations within genes[10], the within-gene locations included in the Annovar distribution were converted into four distinct location categories as follows:

exonic, ncRNA_exonic exonic

UTR3, UTR5, ncRNA_UTR3, ncRNA_UTR5 UTR

intronic, splicing, ncRNA_intronic, ncRNA_splicing intronic

upstream, downstream flanking

For a SNP in multiple categories, the category higher in the above list is chosen only (*e.g*. a SNP that is both in an exon and upstream of the promoter for a non-coding gene is classified as exonic only). Upstream and downstream are defined as within 1kB of the most distal promoter and transcriptional terminators defined in ENCODE; this 1kB distance is the default for Annovar.

In the hypothesis generation phase, each gene was assigned to a single GO ID in the biological function category; when a gene is assigned to multiple categories (as is generally the case), it was instead assigned **only **to the category that was closest to containing exactly 125 genes (with ties broken for smaller categories, so a GO ID with 100 genes would be preferred to a GO ID with 150). Once this was resolved, all GO ids with 50 or fewer assigned genes, along with all intergenic regions, were assigned to a large "OTHER" category.

In the hypothesis testing phase, this mapping to a single GO id is not used. Instead, genes were assigned either to one of the **Group 1 **GO IDs in a group for which our haplotype score had a positive association with eQTL status in the validation/model selection phase (see **Conditional logistic regression **below,) to a **Group 2 **GO IDs for which our haplotype score had a **negative **association with eQTL status in the validation/model selection phase, to GO categories in both groups, or to neither group; eQTLs in intergenic regions were assigned to the group(s) corresponding to the closest flanking genes.

### Conditional logistic regression

Conditional logistic regression, as implemented in the survival package[52] for the statistical programming language R, is used for all statistical model fitting. The notation used in the results closely matches the R syntax, with gene assignments from Annovar (see **Covariates incorporated using Annovar and the UCSC Table Browser**) used as the stratification variable. Each intergenic region is treated as an individual and distinct gene for stratification purposes.

### Meta-analysis of conditional logistic regression results

In the **Conditional logistic regression **above, gene by gene variability plays a significant role. Therefore, in order to identify GO categories in which the Adjusted Haplotype Conditional Entropy reproducibly reported different extremes, the 1-to-1 mapping of genes to GO IDs was permuted 100 times (with genes in the OTHER category, including intergenic regions and genes mapped to GO IDs with fewer than 50 members after 1-to-1 mapping, not permuted). Within each of the 100 permutations, the statistical model (**Model 6**, see Results) was fit to the permuted data.

In both the original data and in each permutation, the following score was summed across the seven data sets:

(5) $$\begin{array}{c} \text{If}\left(\beta >\sigma \right)\beta -\sigma \\ \text{If}\left(\beta <0-\sigma \right)\beta +\sigma \\ 0\;\;\text{otherwise}. \end{array}$$

Where β is the effect size, and σ is the standard error in the effect size. The 100 permutations of 94 such GO IDs produced 9,400 such sums - these were used to produce a 90% confidence interval, and GO IDs in the original data for which the sum was outside of this confidence interval, were continued onto the hypothesis-testing phase.

### Supplementary enrichment tests

As a supplement to the analysis reported in the main text, an additional round of analysis, testing for enrichment of extreme changes in likelihood when models incorporate additional positive selection predictions according to GO IDs, is reported in additional file 1.

## Competing interests

The authors declare that they have no conflicts of interest in relation to this SNP-SIG article.

## Authors' contributions

The original concept was developed by SKH, MS, RMS, KH, DW and WS. Experiments were designed and carried out by SKH, MS, KH, MP, ADJ and AK. All authors contributed towards the writing of the manuscript; all authors have read and approved the manuscript.

SKH^§^: co-designed the new method, performed the analysis, interpreted the results, and co-wrote the manuscript.

MS: co-designed the new method, assisted with analysis and interpretation, and assisted with the manuscript.

RMS: co-designed the new method and assisted with the manuscript. KH: assisted with the analysis and interpretation, and assisted with the manuscript.

DW: assisted with the method design and the interpretation. MP: assisted with the analysis and interpretation.

ADJ: contributed to the analysis, aided the interpretation, and assisted with the manuscript.

AK: contributed to the analysis, aided the interpretation, and assisted with the manuscript.

WS: co-designed the new method and co-wrote the manuscript.

## Supplementary Material

Additional file 1 **See Conditional Haplotype Information and QTLs wTemplate Jan 29 - Supplement**.pdf See Handelmanetal_Figure1.pdf Click here for file
