# Supplementary material for: Conditional entropy in variation-adjusted windows detects selection signatures associated with expression quantitative trait loci (eQTLs)
Source: BMC Genomics. 2015 Jun 18;16(Suppl 8):S8. doi: 10.1186/1471-2164-16-S8-S8 (PMC4480832; doi:10.1186/1471-2164-16-S8-S8)
Supplement: Additional file 1 — See Conditional Haplotype Information and QTLs wTemplate Jan 29 - Supplement.pdf See Handelmanetal_Figure1.pdf [file 1471-2164-16-S8-S8-S1.pdf]

Supplementary Models

These models are fit to the same data sets, using the same conditional logistic regression, as is described in the methods section of the main text.

S0: eQTL ~ MAF + ΔDAF + location
S1: eQTL ~ H|H + MAF + ΔDAF +location
S2: eQTL ~ ΔiHH + MAF + ΔDAF +location
S3: eQTL ~ iHS + MAF + ΔDAF + location

Marginal Probabilities provided to PantherDB

To generate a statistic to be used by PantherDB, the following sum was taken for each gene (as delineated by annovar, see main text) in each data set, from each of the supplementary models above (X = 1 through 3), except for S0:

Eq. S1 Average all eQTLs (Marginal_{SX} – Marginal_{S0}) + Average all non-eQTLs (Marginal_{S0} – Marginal_{SX})

These marginals are reported by the conditional logistic regression, described in the main body of the text, for each of the supplementary models fit to each of the 7 hypothesis-generation data sets (the model containing F_{st} did not converge in the Mangravite data set.) These averages are taken over each gene. This corresponds to the average likelihood gain for each prediction when the new variable is added to the logistic model.

These values are then used for a statistical enrichment test (non-parametric, as implemented in pantherDB) to test for an enrichment of extreme values, which reports p-values. These p-values are then combined across data sets (**NOT** across models) using Fisher's method (i.e., twice the opposite of the sum of the log p-values is tested against a distribution with 2N degrees of freedom, where N is the number of data sets for which a given GO category was present at all.)

Any GO category which in either tail at a one-tailed p-value of less than 0.01 (the same threshold as used in the main text of the paper, albeit for a permutation-based method) is then assigned to either Group 1 or Group 2.

Finally, these Group 1 and Group 2 assignments are tested for an interaction with the predictor as was done in the Hypothesis-Testing phase of the main manuscript (results given in Table S2, S3 and S4). None of the Group 1 vs. Group 2 distinctions are statistically significant; however, both Models S2 and S3 (utilizing ΔiHH and iHS respectively) recapitulate the expect direction of effect, with each unit of log p-value contributing more in Group 2 (GO categories with strong marginal differences in the Hypothesis-Generation data set) than in Group 1 (GO categories with weak marginal differences). On an individual group basis, iHS is a nominally significant predictor of eQTLs in Group 2, while ΔiHH is, paradoxically, a predictor of non-eQTL markers in Group 1.

Supplementary Results, GO Categories

Go enrichnment tests implemented in PantherDB (see main text for references) where used to identify GO categories in which the addition of different positive-selection measures were strong predictors (Group 1), or extremely weak predictors. This resulted in the following GO categories from each of the supplementary models (see table below):

Group 1, H|H (Model S1)
http://amigo.geneontology.org/amigo/term/GO:0034032
purine nucleoside bisphosphate metabolic process
http://amigo.geneontology.org/amigo/term/GO:0033875
ribonucleoside bisphosphate metabolic process
http://amigo.geneontology.org/amigo/term/GO:0033865
nucleoside bisphosphate metabolic process
http://amigo.geneontology.org/amigo/term/GO:0006921
cellular component disassembly involved in execution phase of apoptosis
http://amigo.geneontology.org/amigo/term/GO:0030262
apoptotic nuclear changes
http://amigo.geneontology.org/amigo/term/GO:0006817
phosphate ion transport
http://amigo.geneontology.org/amigo/term/GO:0050832
defense response to fungus
http://amigo.geneontology.org/amigo/term/GO:0044364
disruption of cells of other organism
http://amigo.geneontology.org/amigo/term/GO:0031640
killing of cells of other organism

Group 2, H|H (Model S2)
http://amigo.geneontology.org/amigo/term/GO:0009179
purine ribonucleoside diphosphate metabolic process
http://amigo.geneontology.org/amigo/term/GO:0009135
purine nucleoside diphosphate metabolic process
http://amigo.geneontology.org/amigo/term/GO:0009132
nucleoside diphosphate metabolic process
http://amigo.geneontology.org/amigo/term/GO:0071276
cellular response to cadmium ion
http://amigo.geneontology.org/amigo/term/GO:0046031
ADP metabolic process

Group 1, ΔiHH (Model S1)
http://amigo.geneontology.org/amigo/term/GO:0009451
RNA modification
http://amigo.geneontology.org/amigo/term/GO:0001510
RNA methylation
http://amigo.geneontology.org/amigo/term/GO:0045639
positive regulation of myeloid cell differentiation
http://amigo.geneontology.org/amigo/term/GO:0045637
regulation of myeloid cell differentiation
http://amigo.geneontology.org/amigo/term/GO:0000375
RNA splicing, via transesterification reactions

http://amigo.geneontology.org/amigo/term/GO:0008380
RNA splicing
http://amigo.geneontology.org/amigo/term/GO:0000398
mRNA splicing, via spliceosome
http://amigo.geneontology.org/amigo/term/GO:0000377
RNA splicing, via transesterification reactions with bulged adenosine as nucleophile
http://amigo.geneontology.org/amigo/term/GO:0006396
RNA processing
http://amigo.geneontology.org/amigo/term/GO:0032481
positive regulation of type I interferon production
http://amigo.geneontology.org/amigo/term/GO:0035455
response to interferon-alpha

Group 2, ΔiHH (Model S2)
http://amigo.geneontology.org/amigo/term/GO:0010818
T cell chemotaxis
http://amigo.geneontology.org/amigo/term/GO:0043950
positive regulation of cAMP-mediated signaling
http://amigo.geneontology.org/amigo/term/GO:0048247
lymphocyte chemotaxis
http://amigo.geneontology.org/amigo/term/GO:0007144
female meiosis I
http://amigo.geneontology.org/amigo/term/GO:0032722
positive regulation of chemokine production
http://amigo.geneontology.org/amigo/term/GO:0048513
organ development
http://amigo.geneontology.org/amigo/term/GO:0002483
antigen processing and presentation of endogenous peptide antigen
http://amigo.geneontology.org/amigo/term/GO:0019885
antigen processing and presentation of endogenous peptide antigen via MHC class I
http://amigo.geneontology.org/amigo/term/GO:0019883
antigen processing and presentation of endogenous antigen

Group 1, iHS (Model S1)
http://amigo.geneontology.org/amigo/term/GO:0045069
regulation of viral genome replication
http://amigo.geneontology.org/amigo/term/GO:0002244
hematopoietic progenitor cell differentiation
http://amigo.geneontology.org/amigo/term/GO:0006625
protein targeting to peroxisome
http://amigo.geneontology.org/amigo/term/GO:0072663
establishment of protein localization to peroxisome
http://amigo.geneontology.org/amigo/term/GO:0072662
protein localization to peroxisome
http://amigo.geneontology.org/amigo/term/GO:0043574
peroxisomal transport
http://amigo.geneontology.org/amigo/term/GO:0006182
cGMP biosynthetic process

Group 2, iHS (Model S2)

http://amigo.geneontology.org/amigo/term/GO:0071482
    cellular response to light stimulus
http://amigo.geneontology.org/amigo/term/GO:1901072
    glucosamine-containing compound catabolic process
http://amigo.geneontology.org/amigo/term/GO:0032735
    positive regulation of interleukin-12 production
http://amigo.geneontology.org/amigo/term/GO:0006032
    chitin catabolic process
http://amigo.geneontology.org/amigo/term/GO:0006030
    chitin metabolic process
http://amigo.geneontology.org/amigo/term/GO:1901071
    glucosamine-containing compound metabolic process
http://amigo.geneontology.org/amigo/term/GO:0046902
    regulation of mitochondrial membrane permeability
http://amigo.geneontology.org/amigo/term/GO:0090559
    regulation of membrane permeability
http://amigo.geneontology.org/amigo/term/GO:0032655
    regulation of interleukin-12 production
http://amigo.geneontology.org/amigo/term/GO:0002687
    positive regulation of leukocyte migration
http://amigo.geneontology.org/amigo/term/GO:0002685
    regulation of leukocyte migration
http://amigo.geneontology.org/amigo/term/GO:0002690
    positive regulation of leukocyte chemotaxis
http://amigo.geneontology.org/amigo/term/GO:0002688
    regulation of leukocyte chemotaxis
http://amigo.geneontology.org/amigo/term/GO:0050819
    negative regulation of coagulation
http://amigo.geneontology.org/amigo/term/GO:0050832
    defense response to fungus

Supplementary Hypothesis-Testing Phase (see Tables S2 through S4)

The above groupings are tested for an interaction in the same data set and the same manner as in the main text, except that an interaction with the "Group" designation is added to the predictor which is added to each supplementary model above.

Supplementary Tables

Table S1: Conditional logistic regression model fit parameters in the seven hypothesis-generation data sets.

Model	$\beta_{H\|H} \pm \sigma_{H\|H}$	$\beta_{\|\Delta DAF\|} \pm \sigma_{\|\Delta DAF\|}$	$\beta_{\Delta DAF} \pm \sigma_{\Delta DAF}$	$\beta_{\Delta iHH} \pm \sigma_{\Delta iHH}$	$\beta_{Fst} \pm \sigma_{Fst}$	$\beta_{iHS} \pm \sigma_{iHS}$	$\beta_{MAF} \pm \sigma_{MAF}$	$\beta_{UTR} \pm \sigma_{UTR}$	$\beta_{Intron} \pm \sigma_{Intron}$	$\beta_{Flank} \pm \sigma_{Flank}$
Mangravite 2012										
Mod. 1	Model did not converge									
Mod. 2	0.03 ± 0.001	0.06 ± 0.011	-0.24 ± 0.016	0.002 ± 0.004	n/a	0.05 ± 0.006	1.07 ± 0.014	0.10 ± 0.040	-0.22 ± 0.032	-0.17 ± 0.041
Mod. 3	Model did not converge									
Mod. 4	Model did not converge									
Mod. 5	0.03 ± 0.001	0.08 ± 0.011	-0.23 ± 0.015	n/a	n/a	n/a	× GO	0.09 ± 0.040	-0.22 ± 0.032	-0.17 ± 0.041
Mod. 6	× GO	n/a	0.28 ± 0.014	n/a	n/a	n/a	× GO	0.10 ± 0.040	-0.21 ± 0.032	-0.17 ± 0.041
Montgomery 2010 - Exon										
Mod. 1	**0.04 ± 0.003**	0.16 ± 0.033	-0.25 ± 0.030	*0.01 ± 0.007*	-0.08 ± 0.019	-0.02 ± 0.010	**0.97 ± 0.028**	-0.25 ± 0.068	**-0.83 ± 0.050**	-0.58 ± 0.069
Mod. 2	**0.04 ± 0.003**	0.05 ± 0.020	-0.24 ± 0.030	*0.005 ± 0.007*	n/a	-0.03 ± 0.010	**1.01 ± 0.026**	-0.25 ± 0.068	**-0.83 ± 0.050**	-0.58 ± 0.069
Mod. 3	**0.04 ± 0.003**	0.17 ± 0.033	-0.25 ± 0.030	*0.004 ± 0.007*	-0.08 ± 0.019	-0.03 ± 0.010	× GO	-0.26 ± 0.068	**-0.83 ± 0.050**	-0.58 ± 0.069
Mod. 4	**0.04 ± 0.003**	0.17 ± 0.033	-0.26 ± 0.028	n/a	-0.08 ± 0.019	n/a	× GO	-0.26 ± 0.068	**-0.83 ± 0.050**	-0.58 ± 0.069
Mod. 5	**0.04 ± 0.003**	0.05 ± 0.020	-0.25 ± 0.028	n/a	n/a	n/a	× GO	-0.26 ± 0.068	**-0.83 ± 0.050**	-0.58 ± 0.069
Mod. 6	× GO	n/a	**-0.29 ± 0.025**	n/a	n/a	n/a	× GO	-0.25 ± 0.068	**-0.83 ± 0.050**	-0.58 ± 0.069
Montgomery 2010 - Transcript										
Mod. 1	0.04 ± 0.005	0.22 ± 0.061	-0.19 ± 0.052	0.04 ± 0.014	-0.10 ± 0.033	-0.04 ± 0.018	**0.86 ± 0.050**	*-0.15 ± 0.120*	-0.80 ± 0.088	-0.54 ± 0.122
Mod. 2	0.04 ± 0.005	0.09 ± 0.036	-0.18 ± 0.052	0.04 ± 0.014	n/a	-0.05 ± 0.018	**0.92 ± 0.046**	*-0.14 ± 0.120*	-0.80 ± 0.088	-0.54 ± 0.122
Mod. 3	0.04 ± 0.005	0.24 ± 0.062	-0.20 ± 0.052	0.04 ± 0.014	-0.11 ± 0.033	*-0.04 ± 0.018*	× GO	*-0.18 ± 0.121*	-0.80 ± 0.088	-0.55 ± 0.123
Mod. 4	0.04 ± 0.005	0.24 ± 0.062	-0.19 ± 0.052	n/a	-0.11 ± 0.034	n/a	× GO	*-0.17 ± 0.121*	-0.80 ± 0.088	-0.56 ± 0.123
Mod. 5	0.04 ± 0.005	0.09 ± 0.036	-0.18 ± 0.050	n/a	n/a	n/a	× GO	*-0.17 ± 0.121*	-0.80 ± 0.088	-0.56 ± 0.123
Mod. 6	× GO	n/a	-0.25 ±	n/a	n/a	n/a	× GO	*-0.15 ±*	-0.81 ±	-0.57 ±

			0.045					*0.121*	0.089	0.124
Schadt 2007										
Mod. 1	0.01 ± 0.006	*0.10 ± 0.070*	-0.32 ± 0.067	*0.01 ± 0.016*	*-0.06 ± 0.042*	*0.004 ± 0.023*	**0.96 ± 0.061**	*0.03 ± 0.143*	-0.80 ± 0.113	-0.73 ± 0.157
Mod. 2	0.01 ± 0.006	*0.02 ± 0.043*	-0.30 ± 0.066	*0.01 ± 0.016*	n/a	*0.002 ± 0.023*	**0.99 ± 0.056**	*0.03 ± 0.143*	-0.80 ± 0.113	-0.73 ± 0.157
Mod. 3	0.01 ± 0.006	*0.11 ± 0.070*	-0.32 ± 0.068	*0.01 ± 0.016*	*-0.06 ± 0.042*	*0.005 ± 0.023*	× GO	*0.03 ± 0.144*	-0.80 ± 0.113	-0.73 ± 0.157
Mod. 4	0.02 ± 0.006	*0.12 ± 0.070*	-0.31 ± 0.067	n/a	*-0.06 ± 0.042*	n/a	× GO	*0.03 ± 0.144*	-0.80 ± 0.113	-0.73 ± 0.157
Mod. 5	0.02 ± 0.006	*0.03 ± 0.043*	-0.29 ± 0.065	n/a	n/a	n/a	× GO	*0.04 ± 0.144*	-0.80 ± 0.113	-0.73 ± 0.157
Mod. 6	× GO	n/a	-0.33 ± 0.056	n/a	n/a	n/a	× GO	*0.02 ± 0.144*	-0.80 ± 0.113	-0.73 ± 0.158
Stranger 2007										
Mod. 1	0.02 ± 0.003	0.13 ± 0.031	**-0.35 ± 0.032**	-0.02 ± 0.008	-0.14 ± 0.018	-0.02 ± 0.011	**1.1 ± 0.029**	-0.14 ± 0.070	**-0.55 ± 0.052**	-0.43 ± 0.072
Mod. 2	0.02 ± 0.003	-0.04 ± 0.019	**-0.31 ± 0.031**	-0.02 ± 0.008	n/a	-0.03 ± 0.011	**1.2 ± 0.027**	-0.15 ± 0.070	**-0.55 ± 0.052**	-0.43 ± 0.072
Mod. 3	0.02 ± 0.003	0.16 ± 0.031	**-0.36 ± 0.032**	-0.02 ± 0.008	-0.16 ± 0.019	*-0.02 ± 0.011*	× GO	-0.16 ± 0.070	**-0.56 ± 0.052**	-0.44 ± 0.072
Mod. 4	0.02 ± 0.003	0.16 ± 0.031	**-0.38 ± 0.032**	n/a	-0.16 ± *0.019*	n/a	× GO	-0.16 ± 0.070	**-0.56 ± 0.052**	-0.44 ± 0.072
Mod. 5	0.02 ± 0.003	-0.04 ± 0.019	**-0.33 ± 0.030**	n/a	n/a	n/a	× GO	-0.16 ± 0.070	**-0.56 ± 0.052**	-0.44 ± 0.072
Mod. 6	× GO	n/a	**-0.30 ± 0.026**	n/a	n/a	n/a	× GO	-0.16 ± 0.070	**-0.56 ± 0.052**	-0.45 ± 0.072
Veyrieras 2008										
Mod. 1	0.02 ± 0.003	0.22 ± 0.030	*-0.04 ± 0.028*	-0.02 ± 0.007	**-0.22 ± 0.017**	*-0.01 ± 0.01*	**1.16 ± 0.028**	-0.25 ± 0.064	**-0.67 ± 0.048**	-0.67 ± 0.069
Mod. 2	0.02 ± 0.003	-0.07 ± 0.017	*0.006 ± 0.027*	-0.02 ± 0.007	n/a	*-0.02 ± 0.01*	**1.27 ± 0.026**	-0.25 ± 0.064	**-0.67 ± 0.048**	-0.67 ± 0.069
Mod. 3	0.02 ± 0.003	0.22 ± 0.030	*-0.04 ± 0.028*	-0.02 ± 0.007	**-0.22 ± 0.017**	*-0.007 ± 0.01*	× GO	-0.25 ± 0.064	**-0.67 ± 0.048**	-0.66 ± 0.069
Mod. 4	0.02 ± 0.003	0.23 ± 0.030	-0.06 ± 0.027	n/a	**-0.22 ± 0.016**	n/a	× GO	-0.25 ± 0.064	**-0.67 ± 0.048**	-0.66 ± 0.068
Mod. 5	0.02 ± 0.003	-0.07 ± 0.017	*-0.02 ± 0.026*	n/a	n/a	n/a	× GO	-0.25 ± 0.064	**-0.67 ± 0.048**	-0.67 ± 0.068
Mod. 6	× GO	n/a	*0.02 ± 0.023*	n/a	n/a	n/a	× GO	-0.25 ± 0.064	**-0.67 ± 0.048**	-0.68 ± 0.069
Zeller 2010										
Mod. 1	**0.03 ± 0.002**	0.13 ± 0.019	**-0.28 ± 0.018**	0.01 ± 0.004	-0.08 ± 0.011	0.01 ± 0.006	**0.88 ± 0.016**	*-0.08 ± 0.046*	**-0.43 ± 0.035**	-0.47 ± 0.048
Mod. 2	**0.03 ± 0.002**	0.02 ± 0.012	**-0.26 ± 0.017**	0.01 ± 0.004	n/a	*0.01 ± 0.006*	**0.92 ± 0.015**	*-0.08 ± 0.046*	**-0.43 ± 0.035**	-0.47 ± 0.048
Mod. 3	**0.03 ±**	0.13 ±	**-0.28 ±**	0.01 ±	-0.08 ±	*0.01 ±*	× GO	*-0.08 ±*	**-0.43 ±**	-0.47 ±

	**0.002**	0.019	**0.018**	0.004	0.011	*0.006*		*0.046*	**0.035**	0.048
Mod. 4	**0.03 ± 0.002**	0.13 ± 0.019	**-0.27 ± 0.017**	n/a	-0.08 ± 0.011	n/a	× GO	*-0.08 ± 0.046*	**-0.43 ± 0.035**	-0.47 ± 0.048
Mod. 5	**0.03 ± 0.002**	0.03 ± 0.011	**-0.25 ± 0.017**	n/a	n/a	n/a	× GO	*-0.08 ± 0.046*	**-0.43 ± 0.035**	-0.47 ± 0.048
Mod. 6	× GO	n/a	**-0.25 ± 0.015**	n/a	n/a	n/a	× GO	*-0.08 ± 0.046*	**-0.43 ± 0.035**	-0.48 ± 0.048

Each column gives the effect size on a logistic scale (β) and, gives the standard error in that effect size (σ), for each variable (subscript) from the corresponding conditional logistic model fit (row; for both see text) for being an eQTL stratified by gene or intergenic region. Grey cells are fit on a per-GO term basis (choice results from Model 6 are given in Table 4); black cells are for variables not included in the corresponding model. SNPs in Exons are the default category, so all effect sizes for within-gene SNP location are given relative to the frequency of eQTLs within exons. Bold text indicates an absolute Z-score greater than 10; italicised text indicates an absolute Z-score less than 2 (this may differ from the apparent arithmetic due to rounding).

Table S2: Whole-blood *cis*-eQTL conditional logistic regression results, supplementary approach for H|H (model S1)

Dataset	Number of *cis*-eQTLs	Num. Genes and Regions	Total GTEx Markers	$\beta_{H\|H} \pm \sigma_{H\|H}$
Westra 2012 – Group 1 from S1	2,905	170	25,468	0.007 ± 0.005
Westra 2012 – Group 2 from S1	2,805	127	24,567	0.005 ± 0.005

Table S3: Whole-blood *cis*-eQTL conditional logistic regression results, supplementary approach for ΔiHH (model S2)

Dataset	Number of *cis*-eQTLs	Num. Genes and Regions	Total GTEx Markers	$\beta_{\Delta iHH} \pm \sigma_{\Delta iHH}$
Westra 2012 – Group 1 from S2	12,306	830	116,047	0.026 ± 0.008
Westra 2012 – Group 2 from S2	378	27	1,481	-0.041 ± 0.052

Table S4: Whole-blood *cis*-eQTL conditional logistic regression results, supplementary approach for iHS (model S3)

Dataset	Number of *cis*-eQTLs	Num. Genes and Regions	Total GTEx Markers	$\beta_{iHS} \pm \sigma_{\Delta iHS}$
Westra 2012 – Group 1 from S3	1,485	80	14,408	-0.001 ± 0.027
Westra 2012 – Group 2 from S3	4,294	199	41,941	-0.043 ± 0.016
